# Immediate anaphylaxis after the ingestion of fig fruit (Ficus carica)

**DOI:** 10.1186/2045-7022-5-S3-P167

**Published:** 2015-03-30

**Authors:** Ruperto Gonzalez Perez, Paloma Poza Guedes, Inmaculada Sanchez Machin, Victor Matheu Delgado

**Affiliations:** 1 Allergy Department, Hospital Del Torax, Santa Cruz De Tenerife, Spain

## Rationale

*Ficus carica* is a species of flowering plant in the genus *Ficus*, from the family Moraceae, known as the common fig. Allergic anaphylactic reactions to fig fruit are scarcely reported.

## Methods

A 50 year-old female experienced an episode of respiratory nasal and ocular symptoms (rhinoconjuntivitis) and severe abdominal pain immediately after the ingestion of a fig fruit. No bronchial symptoms were associated.

## Results

Diagnostic work-up showed a positive prick-prick with both fresh raw and dried fig fruit with a total serum IgE of 312 UI/L and a significant serum specific IgE to fig fruit. Co-sensitization to rubber latex and tropical fruits and cross-reactivity between *Ficus benjamina* latex, was also studied and a distinctive serum molecular allergy diagnostic profile (ISAC^®^) was obtained. The patient did not consent to undergo a double blind placebo controlled challenge to fig fruit.

## Conclusion

Herein we present the case of an IgE-mediated allergic anaphylactic reaction to fig fruit showing no cross-reactivity to rubber latex allergens.

## Consent

Written informed consent was obtained from the patient for publication of this abstract and any accompanying images. A copy of the written consent is available for review by the Editor of this journal.

